# Selections and Abstracts

**Published:** 1903-06

**Authors:** 


### SELECTIONS AND ABSTRACTS.

### American Medical Association Meeting in New Orleans.

It has been called the "red-letter meeting of the Association." It was almost the biggest gathering the body has yet held. It surprised many of our own people, who had to be awakened to the importance of so vast an aggregation of special students, engaged in the work for the good of the public health.

Amenities were rife and scientific lore was freely dispensed in every direction. Each section presented a program, in itself sufficient food for the digesting minds of all fortunate enough to follow it. New Orleans has been characterized by an epoch in the history of the advance of medicine in the United States, as expressed by the recent meeting of the National Association, numbering over 2,000 registered physicians, and no one knows how many unregistered. The ladies of New Orleans have marked the occasion with their graciousness and sweet sense of hospitality, while the medical profession has left nothing undone to evidence their own willingness to show the sacrificial spirit in making the stranger guests thoroughly a part of us while they were here.

The aftermath leaves the sense of fullness of thought, not unmixed with the sentiment of certain ideals.

The swan song always carries a few deep notes which have always borne along the soul of the successful achievement, and even if in the old drama the master hand cast the chords of symphony, the current of this thought has always been the skeletal force of the song, and the echo has rung into the minds of all of us who are left behind.

The full power for good of so grand an aggregation of scientific minds can only be felt and known when time has developed it. Like the vibratory force from even a simple source, the many lines of thought emanating from so many different points of view, can only grow in power as they are felt.

The profession has risen by this meeting, risen above certain dogma, above certain crude methods, above certain narrow rules of action, above certain conceptions of ethics, and the future is already budding for a blossoming time of revelation.

The very unit of force for good work inculcated in every man who was here means enough in itself to have justified the meeting, while the seed of the spirit of endeavor was sown everywhere, even among the laity, who touched only the fringe of the inner circle of enlightenment. The glow of pleasure, the essence of education, the throb of a sympathetic spirit of scientific unrest, each has touched the higher planes in the instinct of all, and the New Orleans meeting must pass into history as a momentous occasion pregnant with the idea of a future glory in the medical world and by no means barren of a return in good works.

The 54th annual meeting of the National Association was held in New Orleans May 5, 6, 7, 8, 1903.

We have from month to month related the many preparations made for this gathering, and it is with a high degree of pleasure that we record their successful fruition.

The *New York Medical Journal* remarks this meeting as the "red-letter event of the Association." The editor pays a pretty tribute to the Southern contingent, to whom he credits much original work.

The *New York Medical News* at greater length pays an eloquent tribute to the arrangements and discusses the amount of good of which the meeting will be productive.

It says: "There is no more hopeful index of the brilliant future that is opening for medicine in America than the thorough way in which somewhat abstruse subjects were discussed by Southern members of the Association. It has been realized that there were brilliant lights in medicine at the South, but there can be no doubt after this that the rank and file of the profession, in spite of discouraging conditions in this part of the country in the not very distant past, are fully up to the standard of professional knowledge all over the country, and are, besides, on the crest of the wave of enterprise that is sweeping over the South with regard to progress of every kind.

"Few meetings of the American Medical Association have, we venture to say, been comparable with this in its successful attainment of every end of the annual gathering from the scientific business to the eminently admirable spirit of good fellowship that prevailed. The purely business meetings of the Association seemed to glide easier for the general good-will. The organization of the national body and its close relationship to all lesser societies has now been perfected, and all difficulty over the code seems in a fair way to be settled and a thoroughly united medical profession in America is not far off. The cordial hosts of the Association can take not a little of the credit for the success of the meeting to themselves. The representatives of the American medical profession have learned that the phrase 'Southern hospitality' is no empty name, and that even in the midst of the business bustle of the New South there is a spirit of welcome characteristic of the Southern gentlemen, of whom the country has so long been proud."

The *Journal of the American Medical Association* makes brief editorial mention as follows:

"The session of the American Medical Association just closed has been a success in every respect. The New Orleans arrangements were almost perfect, the general hospitality and feeling was most admirable, and the social functions passed off with delight to all. The Section meetings were above the average in the quality of the papers and the interest in the discussions; the business meetings disposed of matters which have long been a trouble to the profession. The president's address and the orations have already been placed before our readers and need no comment. The attendance was far beyond expectations, and made it one of the largest sessions that the Association has ever held. The general spirit throughout was excellent, and we may say, altogether, that perhaps there has been no session that will leave pleasanter memories with those who attended it."

Rather a trenchant commentary is uttered editorially in the Cincinnati *Lancet Clinic*, curiously presenting a large amount of the opinion held by those whose views have not hitherto been exploited:

"The air was full of mutterings of discontent with the reorganization plans which are being carried on in the Association, many being much opposed, while others are ardent, and ably advocated the change of methods which are being adopted at this time. Criticism was freely expressed in regard to the reorganization methods put in use by the chief reorganizer, who receives a salary of $5,000 a year for his services, which are mainly, at least largely, that of a solicitor for the *Association Journal.* In that way he is supposed to earn his salary. The *Associatian Journal* also sends out from seven to ten or twelve other drummers for the *Journal,* who are authorized by the managers of the Association to offer such terms —cut rates—for new subscribers as to embarrass many other publications. . . Visitors were particularly impressed with the French quarter; it was absolutely unlike any part of any American city on this continent. The newspapers of New Orleans are among the best in the United States, and each has an editorial page of which any newspaper might be proud. The medical profession of New Orleans embraces men of the very finest and highest type to be found in any city, their hospitality being lavish and generous to an extreme. The schools appear to be divided equally between the whites and blacks; if there is a difference observable at all it is in favor of the schools conducted for the negroes alone. No mixed schools are to be found in New Orleans nor should they exist any place else.

"The section work of the Association, which means the scientific, was in every particular quite up to the times, no better or worse than in other years."

So vast a meeting with so many different points of news and of interest would demand large space for an itemized recital; we can only pass a general review in a running commentary of the different functions and purposes accomplished.

Socially the meeting was a success. Not only were men from all sections of the country brought closely together, but the Committee of Arrangements and the ladies of New Orleans can feel satisfied that the St. Charles Hotel Reception, May 5, the Fête Champêtre, May 7, the boat ride, May 8, were successful. The functions at Mrs. Cartwright Eustis's, at Mrs. Maurice Stern's, and the private entertainments given, were sufficient to demonstrate the famed courtesy of the Crescent City, while on every side our guests had the chance to see the best of our Southern women, both young and mature.

The House of Delegates on the several days of their meeting disposed of the business before them:

The Committee on Scientific Research assigned prizes of $100.00 each to Drs. Newton Evans and F. J. Otis, of Battle Creek, Michigan, for work on "Systemic Infection with Blastomycetes"; Mr. G. F. Ruediger, Rush Medical College, Chicago, for work on "Virulent Streptococcus"; Dr. J. P. Moore, Galveston, Texas, for a study on "The Latency and Relapses of Malarial Fever"; Dr. H. E. Weatherill, Philadelphia, for "An Experimental and Clinical Study of Sweat Secretion."

The Business Committee paid a tribute to the Trustees and *Journal;* recommended a new design for the official button; advised postponement of incorporation, which was adopted.

The Code of Ethics and recommended changes were freely discussed, finally resulting in some modifications of the chapters hitherto followed and a change of the title to the "Principles of Ethics." A rather important communication was submitted by the Committee on Medical Education, which was referred.

A suggestion of the President that the Committee on Place of Meeting be made permanent was adopted.

Dental and pharmaceutical members were duly admitted to the Association and a number of minor reports were disposed of.

The Committee on the Prophylaxis of Venereal Diseases was continued and the plan of a National Conference was approved.

On the last day of the meeting, May 8, the following officers were elected:

President—Dr. John H. Musser, Philadelphia.

First Vice-President—Dr. G. C. Savage, Nashville, Tenn.

Second Vice-President—Dr. Isadore Dyer, New Orleans.

Third Vice-President—Dr. C. Lester Hall, Kansas City, Mo.

Fourth Vice-President—Dr. George F. Jenkins, Keokuk, Iowa.

Treasurer—Dr. Henry P. Newman, Chicago.

Secretary and Editor—Dr. George H. Simmons, Chicago.

Trustees—Drs. William H. Welch, Baltimore; Miles F. Porter, Fort Wayne, Ind., and Dr. M. L. Harris, Chicago.

Judicial Council—Drs. F. H. Wiggin, New York; G. B. Gillespie, Tennessee, and D. C. Peyton, Indiana.

Orator on Surgery—Dr. W. J. Mayo, Rochester, Minn.

Orator on Medicine—Dr. George Dock, Ann Arbor, Mich.

Orator on State Medicine—Dr. H. M. Biggs, New York.

Resolutions were adopted thanking the New Orleans Committee of Arrangements and others, as follows:

*Resolved*, That the cordial thanks of this Association be hereby tendered to the fair women and brave men of this historic city, and more especially to the medical profession and the various active and efficient committees through which it has been made effective, for the profuse and charming hospitality which has done so much to make this one of the most pleasant sessions in the history of this Association.

*Resolved*, That thanks be tendered also to the newspapers of the city for the full and impartial reports of the proceedings published, to the transportation companies for reduced rates, and to all others who have contributed to the success and pleasure of this session.

*Resolved*, That especial thanks be extended to the retiring president and to the secretary, for the constant fidelity and distinguished ability manifested in their management of the affairs of the Association, not only during this session, but throughout the year.

The Section Meetings of the Association were full of interest and all were well attended. Those on surgery and medicine taxed the full capacity assigned to them. It is worthy of note that no paper attracted more attention than that of Prof. Stanford E. Chaillé, which was classed by Prof. William H. Welch, of Baltimore, in his address on State Medicine, "as a classic in the history of medicine."

The General Sessions were held, the first at the Tulane Theater, and the rest at the Carondelet Street Methodist Church.

The first day was marked by addresses of welcome on the part of the city by the Hon. Paul Capdevielle, Mayor of the city, on the part of the State by the Hon. Leon Jastremski, and for the citizens of New Orleans by Mr. Henry P. Dart, of the New Orleans Bar, who in a very apt and timely speech touched strongly on the gratuitous services of the medical profession and the future compulsory recognition of this by the State. A response to these addresses was made by Dr. J. T. Witherspoon, of Tennessee, one of the vice-presidents. His speech was full of rich humor and marked by his own appreciation of the fact that he was a Southern man.

The president's address followed, strongly depicting the reasons for medical reorganization in the United States and urging the importance of some restriction of the large annual influx of medical graduates. His argument was logically along the lines of high standards and of legal restriction.

The first, second and third nights of the General Sessions were occupied by the addresses on Medicine, by Dr. J. M. Anders, of Philadelphia; on Surgery, by Dr. F. A. Jonas, of Nebraska, and on State Medicine, by Prof. William H. Welch, of Johns Hopkins University, Baltimore.—*New Orleans Medical and Surgical Journal.*

---

### THE NEGRO.

"Some time ago we had a correspondence in our columns as to the color at birth of the infants of negro parents, both sides expressing their opinion with considerable conviction. It appears that in a recent contribution to the *Revue Encyclopedique*, a German physician, who had spent several years in African Togoland, states that in the equatorial regions the children are born of the same color as European infants. After two or three days the skin turns to a lilac color. Ten days later it becomes of a light chestnut shade, and it is only at the end of three or four months that the skin becomes completely black."—*New York Medical Journal.*

Edelman, *Medical News*, January 31, discussing "The Negro as a Criminal," gives some criminal percentages as follows :

In this country there are 866 criminals to the million among whites; among the blacks there are 2,974.

Considering the arrests made in various cities, are found :

| | | | | | | | |
|---|---|---|---|---|---|---|---|
| Washington, D. C., | population, | white, | 189,457; | total | arrests | 1901, | 12,582 |
| | " | black, | 88,325; | " | " | " | 13,780 |
| Montgomery, Ala., | " | white, | 18,000; | " | " | " | 894 |
| | " | black, | 12,000; | " | " | " | 1,793 |
| Birmingham, Ala., | " | white & black, | 38,415; | " | " | " | 4,030 |
| | | | | " | " | " | 6,600 |
| Louisville, Ky., | " | white, | 148,000; | " | " | " | 3,645 |
| | " | black, | 57,000; | " | " | " | 4,313 |
| Nashville, Tenn., | " | white, | 63,000; | " | " | " | 3,756 |
| | " | black, | 37,000; | " | " | " | 6,081 |
| Atlanta, Ga., | " | white, | 65,000; | " | " | " | 5,784 |
| | " | black, | 38,000; | " | " | " | 11,502 |

In Pennsylvania, according to Professor Starr, of Chicago University, the negro furnishes 16 per cent. of the male and 34 per cent. of the female prisoners, though he constitutes only 2 per cent. of the population.

In Chicago he furnishes 10 per cent. of the arrests, though he forms only 1⅓ per cent. of the population.

The causes of this high percentage are set down as, (1) slavery; (2) ignorance; (3) environment. "Of the 24,272 negro criminals in 1890, 13,138 could neither read nor write, or 57.13 per cent. were absolutely illiterate."

Engelmann, *New York Medical Journal*, February 8 and 15, 1902, considering the "Age of First Menstruation," gives the following points regarding its occurrence in the negro:

His observations are based on a study of more than two thousand individuals observed by himself and others in St. Louis, New Orleans and Baltimore and in Jamaica.

The average age of first menstruation in 2,339 in this country was 14.05. He finds that while the negro of the lowest class menstruates at 14.09, the schoolgirl menstruates at 13.02. Strangely enough, to those who have been accustomed to look for precocity in this respect in residents of the South, the former group was taken from New Orleans, the latter from Boston. This shows the influence of general nervous and mental development and of stimulating surroundings. "The only available statistical study of functional development in the negro is that of Robertson—seventy-seven cases from Jamaica and Barbadoes of the very lowest class." 15.6 years is the average age of pubescence.

A report of the United States Department of Labor shows the following:

MORTALITY OF THE WHITE AND COLORED POPULATION OF SOUTHERN STATES.

(Rates per 1,000 of the Population, 1900.)

| | All Causes. | | Tubercular Diseases. | |
|---|---|---|---|---|
| | White. | Colored. | White. | Colored. |
| New Orleans, La. | 20.7 | 39.7 | 2.38 | 6.13 |
| Mobile, Ala. | 21.5 | 32.3 | 3.08 | 6.09 |
| Charleston, S. C. | 21.4 | 43.1 | 1.65 | 6.21 |
| Savannah, Ga. | 22.6 | 42.2 | 2.64 | 4.94 |
| Richmond, Va. | 18.1 | 32.8 | 2.05 | 3.94 |
| Nashville, Tenn. | 18.3 | 30.6 | 2.11 | 6.29 |

Hoffman, Statistician of the Prudential Life Insurance Company, *Medical Examiner and Practitioner*, October, 1902, says that the insurance of negroes by industrial companies was early recognized to be impossible on the basis of rates charged the white population and, as statutory requirements prohibit differential rates on account of color, few companies make efforts to solicit this class of risks.

In this connection the table given below is of interest:

COMPARATIVE MORTALITY OF THE WHITE AND COLORED POPULATION, CENSUS OF 1900, REGISTRATION AREA, RATES PER 1,000 OF POPULATION.

| Ages. | White. | Colored. | Relative Mortality of the Colored Population.* |
|---|---|---|---|
| 0— 4 | 49.7 | 118.5 | 237 |
| 5—14 | 4.1 | 9.8 | 240 |
| 15—24 | 5.9 | 15.6 | 260 |
| 25—34 | 8.6 | 16.9 | 196 |
| 35—44 | 11.1 | 21.0 | 190 |
| 45—64 | 21.5 | 36.7 | 170 |
| 65+ | 86.0 | 108.6 | 126 |

"This table shows the mortality per 1,000 of population at each age period, together with the relative mortality of the colored population on the basis of the normal death-rate of the whites. It is shown that to every hundred deaths among the white population, ages 5 to 14, there will be 240 deaths among the colored population; at ages 25 to 34 the relative mortality of the colored is 196, and at ages 65 and over, 126. The relative differences in the death-rate are, therefore, greatest at young ages, and less among the element which may be said to represent the results of slavery conditions before the Civil War. In other words, the new generation is shown to be relatively subject to a much higher death-rate than the old, and in the struggle for race survival a type giving evidence of a low degree of vital resistance must needs, in course of time, pass away before the healthier and dominant race.

"The following table embraces a period of ten years, 1893–1902, and includes 29,316 white and 6,134 colored patients.

* Relative number of deaths among the colored population to every 100 deaths among the same number of the white population.

COMPARATIVE MORTALITY PER CENT. OF WHITE AND COLORED PATIENTS BY CLASSES.

Johns Hopkins Hospital, Baltimore, Md.

| | Male Patients. | | Female Patients. | |
|---|---|---|---|---|
| | White. | Colored. | White. | Colored. |
| Medical Cases | 8.6 | 17.6 | 7.3 | 20.1 |
| Surgical | 5.1 | 6,5 | 5.1 | 7.7 |
| Gynecological | — | — | 2.9 | 5.2 |
| Obstetrical | — | — | 1.3 | 1.8 |

Of the medical cases, among males the percentage of deaths was 8.6 for the white and 17.6 for the colored, while for women the difference is even more pronounced. Of the surgical cases the mortality of the colored males was not very much in excess of the death-rate of the whites, supporting the general opinion that the negro stands surgical shock at least as well, if not better, than the white male, but for women the evidence is conclusive that the colored women are less likely to survive surgical operations than white women. In the more complicated gynecological cases the percentage of deaths was much greater among negro women, but ordinary obstetrical cases experienced a death-rate of 1.3 of the white against 1.8 for the colored. This conclusion of a mortality in child-birth among negro women is confirmed by the last census, which gives a death-rate of 5.7 per 10,000, ages 15–49, for the colored, against 3.5 for native whites, 4.5 for those of Irish parentage, and 5.3 for those of German.

"While the special disease liability of the negro has been discussed by many medical writers, but in particular by Reyburn, Corson and Matas, some of the more important results of the experience at Johns Hopkins Hospital may be of interest. The table which follows shows the case mortality percentage of deaths from consumption, pneumonia and typhoid fever, by color and sex of the patients treated during 1893–1902.

"COMPARATIVE MORTALITY FROM SPECIFIC DISEASES.

Johns Hopkins Hospital, Baltimore, Md.
Percentage of Deaths of Cases Treated.

| | Male. | | Female. | |
|---|---|---|---|---|
| | White. | Colored. | White. | Colored. |
| Consumption | 19.2 | 44.5 | 21.4 | 41.6 |
| Pneumonia | 20.7 | 30.2 | 32.0 | 24.3 |
| Typhoid | 6.5 | 10.7 | 6.5 | 11.3 |

French, *Medical Examiner and Practitioner*, December, 1901, says: "The negro in America is also extremely subject to consumption. This seems to date largely from his coming to freedom. Unfitted by generations of slavery to care for himself, and deprived of the care which his master had formerly exercised over him as property, he fell a victim to ignorance, filth and overcrowding, with the diseases which accompany them. In support of this view, it is said that the death-rate of the negro in Charleston, S. C., in 1860 was exactly the same as that of the white man, 12 per 1,000, while in 1895 the white death-rate had increased to 18.7 per 1,000 and that of the negro to 29.1 per 1,000. The deaths from consumption, calculated at ten-year intervals, were as follows: In 1865, whites, 57; colored, 74; in 1875, whites, 54; colored, 132; in 1885, whites, 57; colored, 209; in 1895, whites, 39; colored, 194. Total deaths in 31 years: 1,525 whites, 4,975 colored. Estimated population in 1896: 28,870 whites, 36,295 colored."—*Mississippi Medical Record.*

---

### A FEW SUGGESTIONS ON THE TREATMENT OF STERILITY IN WOMEN.

BY W. O. DAVIS, M.D., NEW YORK.

It is not my intention in this brief article to offer more than a few suggestions on the management of such cases of sterility as can be advantageously treated by the general practitioner. For this reason nothing will be said here of those cases in which this condition is due to mechanical obstacles to copulation and conception which can be removed only by radical operative procedures. This includes all cases in which sterility results from absence of the vulva or vagina, or tumors of these parts, from tumors of uterus, ovaries, or tubes, and from those pronounced uterine displacements which can be rectified only by ventrosuspension or shortening of the round ligaments.

Aside from these conditions, there remains quite a number of cases in which the general practitioner is often able to successfully remove the obstacle to child-bearing. Of course, it must always be borne in mind that in a certain proportion of unfruitful marriages, which is much larger than formerly supposed, the fault lies with the husband. According to Garrigues one out of six husbands is responsible; while Brothers, in a recent article, states that, after carefully examining seventy-two cases of primary sterility and being unable to discover a sufficient cause, he was able to trace the responsibility directly to the husband in fifty of the cases. He concludes that once in every five cases of primary sterility the husband is to blame. Thus it is always important not to lose sight of this fact when we are unable to find any cause of sterility in the woman.

Further we must distinguish between those cases in which a married woman has never conceived and those in which she has previously given birth to children or had miscarriages—the so-called primary and secondary forms of sterility.

When a woman presents herself for treatment of sterility our first question will naturally be: "How many years have elapsed since her marriage or since the birth of her last child?" The answer to the question is of much significance as regards our course of action. According to statistics three fourths of married women become pregnant during the first year of their marriage, and if three years elapse without offspring the chances of impregnation are much reduced (Garrigues).

On the other hand, in women who have previously given birth to children the chances of having other offspring will also proportionately diminish. According to Brothers, whose article is of great interest, in a series of several hundred cases of women under forty years and married more than two years who have remained sterile after miscarriage or childbirth, the chances of further offspring are as follows: Twenty-seven per cent. under thirty years, fifteen per cent. over thirty years, and only seven per cent. between thirty-five and forty years.

These facts must be taken into consideration in making our prognosis. On examination we may find, however, a number of conditions which are directly responsible for the sterility. Of these I would first mention the presence of profuse leucorrhea—due to a vaginal or uterine catarrh. If a vaginal discharge is hyper-acid, this in itself is sufficient to destroy the vitality of the spermatozoa, while the same is true of the cervical mucus, which should normally be of an alkaline reaction. A piece of litmus paper therefore may give us a clue to the treatment.

In cases in which we are able to diagnose the existence of an endometritis this condition must be energetically treated by careful douching, the use of tampons soaked in a ten to twenty per cent. solution of ichthyol in glycerine, or boroglyceride solution, dilatation of the os followed by application to the uterine cavity of iodine and carbolic acid, solution of silver nitrate, or any other remedies which the individual practitioner may select. My preference is nitrate of silver in ten to twenty per cent. solution. This may be neutralized by salt solution. Occasionally curettage will be necessary. If we have to deal simply with cervical catarrh the application should be preceded by the removal of the adherent mucus.

If there is the so-called granular condition of the os, this should be treated by tincture of iodine, acid carbolic, or silver nitrate and scarification.

Caruncle of the urethra sometimes gives rise to so much pain that intercourse is impossible, and the removal of this condition may be sufficient to bring about a cure of sterility. This may be accomplished by application of nitric or chromic acid after previously cocainizing the growth, or snipping it off with scissors and then cauterizing with solid silver nitrate stick.

A prolific cause of sterility are uterine displacements. Although the pessary has gone out of fashion it is occasionally very useful. There are comparatively few women coming under observation in medical practice who will submit to operation for the radical cure of uterine deviations, and in the less severe cases after replacing the uterus the employment of a proper pessary will be sufficient. At the same time the accompanying endometritis must be treated. If, however, laceration of the perineum or a lacerated cervix be present, little can be done without an operation.

Diseases of the ovaries and tubes usually demand treatment by the specialist. Occasionally, after curettage of the uterus and the establishment of free drainage of the cavity, the existing salpingitis is greatly improved or may even be cured. In chronic oöphoritis copious hot-water douches, painting the vaginal vault with tincture of iodine, and tampons of ichthyol and glycerine will be of service.

The local treatment of sterility, to be efficient, must always be supplemented by improvement of the general condition of the patient. In most of these cases there is more or less marked anemia and depression of the nervous system.

If the patient can stand it, there is no better way of stimulating the nervous system than by cold douches, or the cold pack, followed by brisk friction of the skin, preferably on rising in morning. For the anemia a chalybeate should be selected which will not irritate or disturb the digestive organs, as these patients often suffer from constipation and dyspepsia. For this purpose I prefer Pepto-Mangan (Gude), which has the great advantage of palatability, of being rapidly absorbed, and of exhibiting its beneficial effects upon the blood in a short time. Moreover, it does not constipate and does not interfere with the digestion. The administration of small doses of strychnine with Pepto-Mangan (Gude) is often advantageous. It may be given in pill or in the form of a solution in dilute hydrochloric acid, one grain to the ounce, ten minims in a half glass of water before meals. As regards the diet, it need only be said that it should be plain and substantial, avoiding all pastries and other indigestible foods.

In conclusion I would report a few cases of sterility in which I have been able to secure very satisfactory results by the plan of treatment outlined above. These cases, as stated before, comprise only those in which the obstacles to child-bearing can be relieved by methods at the disposal of the general practitioner.

CASE 1.—Mrs. N.———, aged thirty, had been married five years, but never had a child. She was of extremely nervous disposition, and had suffered for some time from attacks of pain in the back and abdomen, shooting in character, and occurring just before the menstrual flow. There was a slight leucorrhea. She had never been averse to having children and had never taken any precautions against conception. Her husband, a vigorous and healthy appearing man, was greatly disappointed, and very desirous of offspring. The patient had always hesitated to consult a physician, as she greatly feared that an operation would be necessary. Moreover, aside from the attacks of pain, she had enjoyed fair health, but lately had been pale and languid. She was also troubled with frequent urination.

On examination the external genitals were found normal. With the patient in Sims's position the cervix was found tilted backward, the fundus forward against the bladder. By bimanual manipulation it could be readily reduced to the normal position, showing that there were no adhesions. The os was somewhat stenosed, but readily admitted the sound. The marked deviation of the uterus seemed to me to sufficiently explain her inability to become pregnant. The indication for treatment, therefore, was to replace the uterus and keep it in position.

After replacing the organ, which was easily accomplished with the fingers in the vagina, a tampon was introduced until a suitable pessary could be provided. Fortunately a Thomas pessary completely held the uterus in place. The patient was also ordered to take copious douches of warm water, to which was added boric acid. Owing to the marked anemia, Pepto-Mangan (Gude) was administered in tablespoonful doses three times daily, and with excellent results. Under its influence the patient's color improved rapidly and she also felt much stronger. The pessary gave rise to no disturbance and under its use the pain in the back disappeared, as well as the frequency of micturition. Some eight months after commencing treatment the patient presented herself with the statement that she thought herself pregnant, as she had some nausea in the morning and her menses had not appeared. An examination made some time later confirmed the correctness of her own diagnosis. She went through practically a normal pregnancy and was delivered by me of a healthy male infant.

Case 2.—Mrs. M———, aged twenty-eight, had been married seven years, and had not enjoyed good health since a miscarriage during the second year of her married life. She complained of dragging pain in the back and had some leucorrhea. She was troubled with indigestion and constipation. Upon examination the uterus was found somewhat enlarged and tender. The os was slightly eroded. The local treatment consisted in copious douches of warm water, warm sitz baths, application of silver nitrate to the eroded surface, and tampons soaked in ichthyol-glycerine. As the patient was very anemic, I gave her Pepto-Mangan (Gude) in tablespoonful doses, three times daily, and kept her bowels regular with cascara.

The patient was much improved in general health and the leucorrhea greatly diminished. The uterine tenderness also decreased and menstruation became less painful. To effect a radical cure it was now thought better to resort to curettage of the uterus after thorough dilatation of the cervix. An application of iodine was then made to the uterine cavity and a strip of gauze left in the cervix for drainage. This was followed by a boroglyceride tampon in the vagina. In the course of two months her condition became markedly changed for the better. I then lost sight of her, but later she returned and engaged me for her confinement which she expected shortly. Although more than four years had elapsed since the miscarriage, the removal of the endometritis, from which she had suffered, also cured her of her sterility.

CASE 3.—Mrs. B——. This was rather a peculiar case. The patient was thirty-four years of age and married four years. She stated that both she and her husband desired offspring, but intercourse had been attended with so much pain and discomfort that it had been abstained from for some time. Urination was also painful. Examination disclosed the presence of a caruncle of the urethra, which was snipped off with scissors and the base cauterized with nitric acid. Her general health, which had been impaired by the constant pain, was greatly benefited by the use of Pepto-Mangan (Gude) and strychnia, as well as by a protracted trip to the country. That the caruncle was the cause of the sterility was shown by the fact that later she became pregnant and went through a normal delivery.—*International Journal of Surgery.*

---

### THE THERAPEUTICS OF EPILEPSY.

BY G. H. FRENCH, A. M.,
Curator and Department of Biology and Physiology.

In one sense epilepsy is not a disease, but a symptom of some diseased or irritated condition of some part of the body. Such reflex spasms in the epileptic are possible, because he is a neurotic whose temperament is such that almost any form of irritation in him causes spasms that would not be manifested in another with a different temperament. The writer has had two cases reported to him where a tapeworm was the cause of epilepsy and in which a removal of the tapeworm cured the cases. But it is well known that it is the rare exception for epilepsy to be associated with this parasite. The writer has known of five cases where the irritation causing the spasms was in whole or in part a small intestinal parasite, the larvæ of a fly related to the horse bot fly, and which has been named *gastrophilus epilepsalis*. In two of these cases, uncomplicated, a removal of the parasites cured the boys. In a large number of cases the writer has found constipation with its resultant autotoxemia a factor or source of irritation, and often associated with this is lack of elimination by the kidneys. The genital organs come in for a large share of the trouble, the irritation of these parts dependent on various causes.

In these peripheral cases of epilepsy, where the source of irritation can be found and the cause removed, the most of them may be cured by such removal, a proper treatment of the nervous system for the spasm habit, and proper attention to diet and hygiene. There is quite a class of cases where the brain and spinal cord are the seats of irritation in which a cure is very doubtful. The writer has had a number of cases reported to him where epilepsy followed a sunstroke, and one following a severe case of whooping-cough. The latter finally died of meningitis. At present I can see no encouragement in this class of cases.

From the above it will be seen that the first thing is to treat whatever is found, after a careful examination, that can act as a source of irritation to cause or keep up the spasms. Second, to treat the nervous system. This should have two objects; to correct the spasm habit and to build up the system so that after a time any slight irritation may not cause it to revert back to this habit again. In this I would say don't give bromides, for they depress instead of build up. In many cases the active principle of verbena hastata, verbenin, has served a good purpose as an antispasmodic and nerve tonic. In most cases the triple arsenates with nuclein are indicated, and also some form of heart tonic. Of these, cactin or digitalin seem to give better results than some of the others.

One writer says an epileptic is a gourmand; and there is reason to believe that in most instances this is true. Only so much should be allowed as is necessary to maintain the system, and in many cases digestion may be needed for a time with this amount. As a general thing, a vegetable diet with very little salt, and a free use of fruits and fruit juices is preferable. I would not replace the salt with bromides, for I would not use the bromides in these cases in any form.

Finally, a large amount of exercise and out-of-door life should be encouraged.—*St. Louis Medical Era.*

---

### THE AMERICAN CONGRESS ON TUBERCULOSIS FOR THE PREVENTION OF CONSUMPTION.

ATLANTA, GA., U. S. A., February 18, 1903.

*To Members of Aemrican Congress on Tuberculosis:*

GENTLEMEN:—Dr. Lewis authorizes the following statements: Dr. Chas. O. Probst, of Columbus, Ohio, has been appointed a member of the Executive Council, Dr. Chas. Wood Fassett, of St. Joseph, Mo., has been appointed 5th Vice-President. The next meeting will be held in St. Louis, Mo., U. S. A., July 18th to 23d, inclusive, 1904. The work of organization is being pushed as rapidly as possible. To facilitate this the Congress has been granted a charter, thus making it a legal body and by this means greatly facilitating the work of reorganization on the lines mapped out at the last meeting, when it was decided that a radical reorganization should be completed by the officers elected. For the purpose of completing the organization of the International or World's Congress on Tuberculosis on strictly ethical lines, the following well-known physicians have been asked to serve on an Advisory Committee to assist the council in perfecting plans for the meeting. All have accepted and a large number will be added to this list: Dr. R. M. Reilley, Surgeon-General U. S. Army; Dr. J. N. Hurty, Sec'y State Board of Health, Indiana; Dr. H. Beaumont Small, Sec'y Canadian Ass'n for Prevention of Consumption;

Dr. Geo. W. Webster, Pres. State Board of Health, Illinois; Dr. A. G. Young, Sec'y Maine State Board of Health; Dr. J. B. Maxwell, Mt. Carmel, Ill; Dr. Thos. F. Harrington, 128 Merrimack St., Lowell, Mass.; Dr. Willis F. Westmoreland, Atlanta, Ga.; Dr. Henry D. Holton, Pres. American Public Health Ass'n; Dr. J. C. Shrader, Sec'y State Board of Health, Iowa; Dr. A. C. Bernays, St. Louis, Mo.; Dr. Irving A. Watson, President N. H. Medical Society; Dr. J. A. Egan, Sec'y State Board of Health, Illinois; Dr. P. M. Rixey, Surgeon-General U. S. Navy; Dr. H. M. Bracken, Sec'y State Board of Health, Minnesota; Dr. J. Harris Pierpont, Pres. Florida State Medical Association; Dr. Chas. Hicks, Pres. Georgia State Medical Association; Dr. W. A. Evans, Pres. Chicago Medical Society; Dr. E. M. Reading, 6501 Kimball Ave., Chicago, Ill.; Dr. J. B. Walker, Effingham, Ill.; Dr. H. H. Stone, Phenix Arizona; Dr. Wm. Oldright, Toronto, Canada; Dr. Chas. O. Probst, Sec'y American Public Health Ass'n.; Dr. Benj. Lee, Sec'y State Board of Health, Pennsylvania; Dr. P. H. Bryce, Sec'y Provincial Board of Health, Toronto; Dr. Frank Paschal, Sec'y Texas State Board of Health; Dr. Manning Simmons, Pres. South Carolina Medical Society; Dr. Donald Campbell, Butte, Montana. The other committees will be named as soon as they can be selected. It is earnestly requested that the Secretary be informed of the date of all meetings of medical societies, names of officers of same and other information that would aid him in his work.

Respectfully,

GEORGE BROWN, Sec'y.

---

### BUNIONS AND THEIR TREATMENT.

The tendency in the treatment of bunions is to do too much or too little, although, perhaps, the latter fault is the prevailing one.

The common site for the beginning of bunion formation is at the bursa on the inner surface of the head of the first metatarsal bone, and a tight shoe is usually responsible for the bursitis which represents the first stage in the development of the deformity. Incidentally the tight shoe is apt to crowd the phalanges of the great toe toward the outer side of the foot, but this, while adding to the deformity, is probably not an essential part of the disease, although it is usually classed as such.

With the progressing bursitis three important changes take place:

1. The synovial sheath of the bursa becomes thickened and hypertrophied in its attempt to secrete enough synovia to protect the region.

2. We have the development of a bony protuberance by hyperostosis beneath the bursa.

3. This change consists in the formation of a thick, horny layer of cuticle similar to an ordinary corn.

Aside from the local pain at the site of the bunion caused by pressure effects on the hypertrophy, we have a secondary cause for inflammation in many cases due to the disinclination of the patient to walk or exercise sufficiently to transform the proteids in his food, and lithemia naturally develops.

If we have a gouty form of uric acid inflammation developing as a result of the lithemia, the patient may suffer from podagra in addition to the results of pressure.

The common forms of palliative treatment, consisting in the use of perforated plasters and removal of the horny layer of cuticle, are not very efficient because of the hypertrophied structure beneath, which will not disappear under anything short of mechanical means.

The older operations aimed at removing the entire head of the first metatarsal bone, and this commonly resulted in a stiff joint which was in itself a source of much discomfort. If we did not have a sufficiently close union to give a stiff joint, the sharp edge of the metatarsal bone, resulting from exsection, would sometimes cut into the tissues of the sole of the foot, so that it becomes necessary to exsect the heads of all of the metatarsal bones.

As a matter of fact a very simple operation will suffice to give relief in many cases of bunions.

*Operation.*—A longitudinal incision, not more than half to three-quarters of an inch in length, along the line of the inner surface of the extensor tendons, exposes the site of the hyperostosis, and a sharp chisel separates the button of the bone readily from the head of the metatarsal bone. The open bursa can then be trimmed out with a pair of scissors without difficulty, and when the wound is sutured and the skin pressed against the surface of bone from which the button was removed, it becomes quickly adherent and the bunion is at an end.

This treatment will suffice to give comfort in most cases and no further operative work is necessary, but if the patient wishes to have the phalanges of the great toe placed in normal line with the metatarsal bone it is necessary to divide the external ligament of the metatarso-phalangeal articulation. This can be done subcutaneously, but in doing so we cannot avoid cutting a pretty large branch of the dorsalis pedis artery, as a rule.

Hemorrhage from this cut artery we can usually disregard if a small, round, gauze pad is strapped firmly down upon the dorsum of the foot at this point beneath a light splint. We must inform the patient, however, that sometimes it becomes necessary to enlarge the incision and ligate the artery. I have not been obliged to do this as yet, but have had to exercise a certain amount of skill in making proper application of pressure.

After the simple operation of removing the button of bone, and the thick bursa, very little further treatment is required, and the patient may be allowed to walk in from ten to twelve days.

In cases, however, in which it has been thought desirable to correct the hallux valgus by division of the external lateral ligament, we must apply a splint to be worn inside the shoe, which will hold the phalanges in place for several months. A very good splint is made by hammering a piece of sheet copper into the shape of the sole of the foot, and turning up a flange which corresponds to the external surface of the great toe. A special stocking is then made with a separate compartment for the great toe, so that the flange from the splint can be interposed between the great toe and the next one.

Very few patients are more grateful than the ones upon whom we operate for the cure of bunions, and it does not seem to be generally known by the laity that we can remove this deformity.

Not long ago, while in a country store, one of the old inhabitants who occupied a cracker-box, said to me—without knowing that I was a physician—that no one could cure bunions, and remarked very impressively that he would give $100 to any one who could cure his. I told him that the usual price for curing them was about twice what he offered, but that it could be done very easily and for nothing at all, if he was not able to pay what the services were commonly considered to be worth.—*Robert T. Morris, M.D., in International Journal of Surgery.*

---

### SOME PHASES OF INFLAMMATION OF THE APPENDIX.

Frederick Treves states that the greater proportion of cases of appendicitis recover spontaneously, and it is probable that the general mortality of the disease—if examples of all grades be included—is not above five per cent. Operations carried out during an acute attack are attended with a risk to life which is considerable, and which is probably expressed by a mortality of over twenty per cent. Certain hospital records and collections of cases appear to place the death-rate even higher than this. It must be remembered that relapses may occur after operation carried out during the acute stage. The removal of the appendix during the quiescent period is attended with a very trivial risk, which may be expressed by a mortality of one in 500. The writer thinks that our knowledge of the pathology of the disease and its general mortality will not sanction the practice of opening the abdomen in every case of appendicitis as soon as the diagnosis is established. Immediate operation is demanded, at the earliest possible moment, in all ultra-acute cases. These include those very hopeless examples which present from the onset the phenomena of intense infection, and in which it is evident that a very large dose of poison has suddenly been introduced into the system. Death may occur in thirty-six or forty-eight hours. There are also included cases in which the symptoms are as acute as those attending perforation of ulcer of the stomach. The writer does not believe that these ultra-acute cases are difficult of recognition. Immediate operation is demanded in every instance in which there is reasonable suspicion that suppuration has taken place. In cases outside of those above named, the question of operation may be kept in abeyance for the first few days after the attack, and may usually be left open for decision until the fifth day or after. The great majority of cases of appendicitis recover spontaneously without either an operation or the formation of an abscess; the ultra-acute cases are actually rare, and relatively to the whole mass of examples of all degree suppuration may be said to be uncommon. There are certain cases of adults in which the attack would appear to be led up to by gross deviations from normal food-taking. For example, those individuals who have no teeth, those who "bolt" their food, those who overeat, those who constantly eat indigestible food, and those who neglect their bowels. If these errors be corrected there may be no repetition of the attack. Removal of the appendix is to be recommended in chronic appendicitis, in those cases in which there are no actual attacks, but in which there is abiding discomfort in the right iliac fossa with exacerbations of uneasiness. The removal of the appendix is not a panacea for all ills, nor even for all those manifold pains which seize upon the lower segment of the abdomen.—*Ex.*

---

### HOW NOT TO BE NERVOUS

Is the topic chosen by Dr. Patrick for his address given before the Mississippi Valley Association, and we rise to remark that it would be hard to find in another ten pages as satisfactory an etiology of American nervousness as that set forth by Dr. Patrick in his charming address. No other American medical writer, not excepting William Osler or Weir Mitchell, wields as graceful a pen as Dr. Patrick; but even better than literary finish is the hard common sense of his address. Dr. Patrick is a comparatively young man but parents and teachers and pediatricians may well ponder long what he has to say about the "centripetal developed" American child and the nervous pitfalls which inevitably lie before such. "To force mature functions from an immature organism is to violate the virginity of Nature, a crime daily committed in the home and in the school, to be expiated in the sick-room, the sanitarium or the asylum" is a sentence worthy to be committed to memory by every one who has been committed to the care or teaching children.

The only just criticism that occurs to us in reading this charming brochure is that etiology and prophylaxis have been discussed at the expense of therapy.

The victim of nervousness knows as a rule only too well what is the matter and why it is so. Like Coleridge they can say "I suffer from an impotence of volition not of the intellectual faculties. You bid me rouse myself, go bid a man paralyzed in both arms to rub them briskly together and that will cure them." We wish Dr. Patrick had told us more in detail how to set the paralyzed will at work. Perhaps he has told us all that can as yet be said when he points us clearly to the causes of American nervousness, viz., misdirected energy, misplaced worry, longing for baubles and the fighting of phantoms. True, O King, but how to dispel these phantoms and most of all that baseless fear which in one form or another enters into the make-up of nearly every sort of nervousness. These things are too many for the average practitioner, but while groping for wider knowledge it is helpful both to the nervous doctor and his more nervous patients that they should take Dr. Patrick's advice and strive not for the unattainable, regret not the unalterable, live with reason, play joyously and love the good and thy neighbor as thyself.—*Chicago Clinic.*

---

### THE CRUELTY OF FOIE GRAS.

The sentimentalists who devote so much energy towards the suppression of experimental scientific research conducted upon the lower animals will find an abundant harvest of absolute wanton cruelty on all hands if they care to look for it. How many anti-vivisectionists, we wonder, eat foie gras? Do they know that it is made of the diseased livers of geese which are deliberately brought to death's door by treatment that is diabolically cruel? The unfortunate birds are cooped up indoors in boxes so arranged that the head alone can be moved. They are then crammed with a rich diet which is forced down their gullet. Under these circumstances the liver quickly becomes affected, and in three months has attained an enormous size from fatty degeneration. The larger the liver the more successful the process. The most valuable livers are those of a green tint; that is to say fatty livers impregnated with bile pigments. The center of this trade is Strasburg, which sends out annually about £150,000 worth of this delicacy. A recent petition to the civic authorities of London to exclude foie gras from the banquet recently given to the Prince of Wales has excited the liveliest alarm among the merchants of Strasburg, inasmuch as, after Paris, England is the next best customer. Three months of forced feeding is required to bring the unfortunate birds to the proper pitch of organic degeneration, so that their livers may tickle the palates of fat gourmands. Of a truth any anti-vivisectionist who eats foie gras is committing an act of farcical incongruity. On the one hand he is eating a toothsome morsel procured by a course of prolonged torture practiced upon a harmless domestic fowl, while on the other he is railing at scientific men whose aim in experimentation is the highest conceivable —namely, the alleviation of suffering among mankind. Meanwhile Strasburg flourishes and science is tied hand and foot in the United Kingdom.—*Med. Press and Circular.*

---

### SURGICAL HINTS.

A plaster cast can easily be removed with a knife if the line of incision has been beforehand well saturated with ordinary vinegar, which softens the plaster.

The ordinary washing soda solution in which instruments are boiled will entirely ruin aluminum instruments, and hence should not be used for sterilizing them.

Plaster of paris dressings that are liable to be moistened by secretions from a wound, as, for instance, in the treatment of compound fractures or of excisions, where a fenestrum is made, can be nicely protected by painting the exposed parts with melted paraffin.

In cases of injury to the brain, operation is never permissible unless there is distinct evidence of the fact that some condition exists which trephining will probably relieve. Even a bullet or larger body is not to be sought for unless it gives clear symptoms of compression or irritation, or unless hemorrhage is going on.

In children prone to the development of so-called scrofulous glands of the neck, it is a mistake, unless they rapidly disappear under the influence of local and general treatment, to await the formation of abscesses. The latter cause more marked cicatricial deformity than does proper incision with enucleation, and there is always a possibility of systemic infection from the abscess.

It is well to remember that cervical adenitis is often caused by the presence of pediculi of the scalp. It is more frequent in girls than in boys, owing to the greater length of the hair, which harbors a larger number of insects, thus causing more irritation and hence more eczema due to scratching. In looking for pediculi they are most easily found in girls in the hair growing lowest at the back of the neck, and in boys in the lank hair back of the ears.

In a case of gonorrhea in the male, a chill occurring without swelling of the external organs strongly suggests the possibility of prostatic abscess. Always examine by the rectum, and if the gland is large and tender, and hard though elastic, little time should be wasted before opening the perineum. The old method of opening through the rectum with a trocar is a bad one, because it is unclean and liable to result in the formation of urethro-rectal fistula, and because the abscess cannot be incised and drained as widely as by the perineal route.—*International Journal of Surgery.*

---

### PRESENT STATUS OF THE PESSARY.

Davenport says that the pessary, the use of which twenty-five years ago was the sole method for the treatment of uterine displacements, has, with the development of surgical methods, suffered a temporary neglect. It is now regaining its position to some extent, and the indications for its use are better understood and are upon a more scientific basis. In uncomplicated cases in young women who have not had treatment, operation should be advised. Operation is the only method which holds out prospect of cure in cases complicated with lacerations, enlarged uterus, or much prolapse. In other cases, especially when the uterus is small, when symptoms have been present but a short time, and particularly if they are associated with neurasthenia, treatment by pessary will often result in a cure, probably in one half of such selected cases. Even when cure cannot be hoped for by pessary its temporary use is often of value in relieving symptoms and in aiding to restore the general health. Some principles governing the use of the instrument are given. In case of retroversion or flexion, always replace the uterus before adjusting the support, as the pessary cannot be relied upon to do this. In fitting a support choose one which fits exactly, if possible; but if not, have it rather too small than too large. The patient should be seen at regular intervals while she is wearing a pessary, and when deemed advisable to make an attempt to go without it, it should not be removed at once, but a smaller one substituted, to be worn a month and then a still smaller one, which may then be finally removed.—*Boston Medical and Surgical Journal.*

---

### ENURESIS.

(F. H. Darby, M.D., Cincinnati *Lancet-Clinic*, March 28, 1903.) The condition is said to be most prevalent between the ages of three and twelve years; and occurs more frequently in males. The author believes that the mind plays a more important part than is generally conceded and reviews at length the various causative factors.

Care should be exercised in the diagnosis to separate those of nervous origin from those due to purely local causes. Attention is called to the examination of the rectum for hemorrhoids and ascarides, the chemical examination of the urine, the presence of calculi and the correction of digestive disorders. These children should not be punished, and where possible should have their sanitary surroundings improved by being removed from institutions and placed in well regulated families.

The drug treatment is to be adapted to the needs of the child and includes tonics, such as iron, strychnia, cod-liver oil, with plenty of outdoor exercise for the debilitated cases. In chronic cases arsenic, cod-liver oil, quinine, iron and cold baths. In hyperesthesia of the vesical neck minute doses of tincture of cantharides are useful.

In the use of belladonna the writer insists, first that the drug shall be of a good quality and, second, that it be pushed to the full tolerance of the patient.—*St. Paul Med. Jour.*

---

### BASIS FOR RECIPROCAL MEDICAL REGISTRATION.

That for the purpose of establishing medical reciprocity among the States composing it, the American Confederation of Reciprocating Examining and Licensing Medical Boards does hereby agree to the following propositions as a basis of reciprocal medical registration.

(a) That as a prerequisite to reciprocal registration, the applicant therefor shall file in the office of the Board of the State of which he is a licentiate such evidence as will enable the said board to certify that he is of good moral and professional character.

Such certificate shall be filed with his application for reciprocal registration in another State.

*Qualification No. 1.*—(b) That a certificate of registration showing that an examination has been made by the proper Board of any State, on which an average grade of not less than seventy-five per cent. was awarded, the holder thereof having been at the time of said examination the legal possessor of a diploma from a medical college in good standing in the State where reciprocal registration is sought, may be accepted, in lieu of examination, as evidence of qualification; provided, that in case the scope of the said examination was less than that prescribed by the State in which registration is sought, the applicant may be required to submit to a supplemental examination by the Board thereof in such subjects as have not been covered.

*Qualification No. 2.*—(c) That a certificate of registration or license issued by the proper Board of any State may be accepted as evidence of qualification for reciprocal registration in any other State; provided, that the holder thereof was, at the time of such registration, the legal possessor of a diploma issued by a medical college in good standing in the State in which reciprocal registration is sought, and that the date thereof was prior to the legal requirement of the examination test in such State.

